# Plasma-Assisted Synthesis of Surfactant-Free and D-Fructose-Coated Gold Nanoparticles for Multiple Applications

**DOI:** 10.3390/ma15217579

**Published:** 2022-10-28

**Authors:** Hafiz M. Yasin, W. Ahmed, N. U. Rehman, Abdul Majd, Mohammad Alkhedher, ElSayed M. Tag El Din

**Affiliations:** 1 Plasma Physics Laboratory, Department of Physics, COMSATS University, Islamabad 45550, Pakistan; 2 Materials Laboratory, Department of Physics, COMSATS University, Islamabad 45550, Pakistan; 3 Department of Physics, University of Gujrat, Gujrat 50700, Pakistan; 4 Mechanical and Industrial Engineering Department, Abu Dhabi University, Abu Dhabi 111188, United Arab Emirates; 5 Electrical Engineering Department, Faculty of Engineering & Technology, Future University in Egypt, New Cairo 11835, Egypt

**Keywords:** AuNPs, SERS, catalytic degradation

## Abstract

The excellent optical properties of gold nanoparticles (*AuNPs*) make them promising for numerous applications. Herein, we present a facile synthesis of both surfactant-free (SF−AuNPs) and non-toxic D-fructose (*DF*)-coated gold nanoparticles (DF−AuNPs) via the plasma–liquid interactions (*PLIs*) method. Moreover, we demonstrate that both SF−AuNPs and DF−AuNPs are potential candidates for trace detection via surface-enhanced Raman scattering (SERS) and catalytic degradation of toxic dyes. However, SF−AuNPs have superior SERS and catalytic performance compared to the DF−AuNPs due to their surfactant-free nature. Moreover, SF−AuNPs have also been shown to quench the fluorescence of analyte molecules, making their SERS-based trace detection more efficient. In particular, SERS enhancement of rhodamine 6G (R6G) and catalytic reduction of a toxic dye methylene blue (MB) have been explored.

## 1. Introduction 

Gold nanoparticles (AuNPs) have attracted great interest owing to their unique properties such as stability, non-toxicity, and surface plasmon resonance (*SPR*), which give rise to interesting optical properties in the visible and near-infrared (IR) regions [1,2,3,4]. These properties make them attractive for applications in catalysis [5,6], sensing [7,8], photodynamic therapy [9,10], drug delivery [11,12], corrosion resistance in electrical connectors [13,14], visible and infrared shielding [15], photothermal therapy [16,17] and so forth. However, the majority of applications require pure, stable and well-defined AuNPs [18]. Therefore, the synthesis of pure, stable, reproducible, and monodispersed AuNPs has attracted great research attention. The synthesis methods employed for this purpose include top-down methods such as laser ablation of solid targets, electrolysis, ball milling, aerosol techniques, and bottom-up techniques such as chemical reduction methods, biological methods, and PLI techniques [19,20,21]. However, most of the top-down methods work either at higher temperatures or at high speeds, which affect the purity and quality of metallic nanoparticles [22,23]. Similarly, some of the bottom-up techniques such as chemical or biological methods require reducing agents for the reduction of gold precursors in a solution. These chemicals may be toxic, but even when they are non-toxic, they affect the purity as well as the application of AuNPs towards sensing and catalysis [24,25]. 

The plasma–liquid interaction (PLI) technique employs the atmospheric pressure microplasma generated above the liquid surface. It is suitable for the synthesis of pure and surfactant-free metallic nanoparticles, as it avoids the usage of reducing agents, and plasma species such as ions, UV-radiation and electrons are utilized for reduction purposes [26]. However, due to the surfactant-free surface, the synthesized *NPs* are not very stable and have a shorter shelf-life. During wet chemical synthesis, different conventional capping agents such as cetyl-trimethylammonium bromide (CTAB), sodium citrate, etc., have usually been employed as a stabilizer to improve the stability of nanoparticles and control their growth kinetics [27,28]. However, the majority of these conventional capping agents are either toxic or have a bigger size that affects the application of *AuNPs* as well as other properties such as sensing and catalysis [29,30,31,32]. For example, for excellent SERS detection capability, the analyte should have a close approach to the surface of AuNPs. This is because, in the SERS technique, the Raman peaks of the target molecules (analytes) are mainly enhanced by the high near field of AuNPs, the metal nanoparticles originating from SPR. Larger molecular stabilizers act as a spacer and keep the analyte away from the surface of the AuNPs, which reduces their SERS capability. Therefore, the larger the size of the stabilizer, the smaller will be the SERS capability of AuNPs. If the size of the capping agent is greater than a couple of nanometers, it can also enhance the featureless fluorescence signal of the analyte [33]. As the Raman cross-section is usually 8 to 10 orders of magnitude lower than the fluorescence cross-section, Raman peaks under such conditions are overshadowed by the fluorescence background [34,35]. Similarly, for excellent catalytic activities of AuNPs, the reactants should be able to adsorb on the surface of the *AuNPs*, which is compromised when the surface of the *AuNPs* is covered with the surfactant molecules. 

The sensing, SERS, catalytic, and biomedical applications of functionalized and unfunctionalized *AuNPs* synthesized by plasma have been explored by a number of researchers. Wang et al. carried out the synthesis of *AuNPs* using atmospheric pressure microplasma [36]. They showed that the stirring mode, stabilizer concentration, and discharge power all affect the final size and size distribution of the synthesized *AuNPs*. By tuning the reaction parameters, the size of the AuNPs was tuned from about 11 nm to 53 nm. The synthesized AuNPs showed excellent capability for the detection of cardiac troponin I [36]. Li et al. employed microplasma-synthesized *AuNPs* with the size ranging from 4 to 12 nm for SERS-based trace detection of methylene blue [37]. They obtained enhancement factors of the order of 10^8^. For the catalytic degradation of organic dyes, usually the composites of *AuNPs* with other materials are employed. For instance, Yang et al. used plasma to fabricate composites containing 10 nm *AuNPs* uniformly distributed within the reduced graphene–oxide matrix and employed them for the catalytic reduction of 4-nitrophenol to 4-aminophenol [38]. Liu et al. fabricated the hybrid structures of carbon nanotubes with *AuNPs* of varying size ranging from 12 to 40 nm and used them for green oxidation of silanes [39]. With regard to biological applications, Nguyen et al. demonstrated that polydopamine-functionalized *AuNPs* synthesized by plasma, having an average size of 54 nm, were excellent for anti-cancer applications due to their polydopamine functionalization [40]. However, despite the previous work, a comparative study of the applications of functionalized and unfunctionalized *AuNPs*, synthesized by *PLI*, is still missing. It would be interesting to explore if the surfactant-free nature of *AuNPs* has any impact on their applications. In the present study, a PLI technique was employed for the synthesis of surfactant-free gold nanoparticles (SF−AuNPs) and D-fructose (*DF*)-coated *AuNPs* (DF−AuNPs). Both *NPs* were used for SERS-based molecular trace detection and catalytic degradations of toxic dyes. The SF−AuNPs clearly showed superior SERS-based trace detection and catalytic reduction capabilities compared to DF−AuNPs, as there was no barrier for the analyte to reach close to the particle’s surface. 

## 2. Experimental Section

### 2.1. Materials and Solution Preparations

For the synthesis, chloroauric acid $\left(HAuCl_{4}>99.99\%\right)$, DF $\left(C_{6}\;H_{12}\;O_{6}\right)$, rhodamine 6G (R6G), and methylene blue (MB) were used. $HAuCl_{4}$, R6G, and MB were purchased from Sigma-Aldrich while D-fructose (DF) was purchased from Avonchem. The utilized chemicals were used as received, and no further purification was performed. Deionized (DI) water was used to prepare all solutions. 

### 2.2. Experimental Setup 

**Figure 1:**
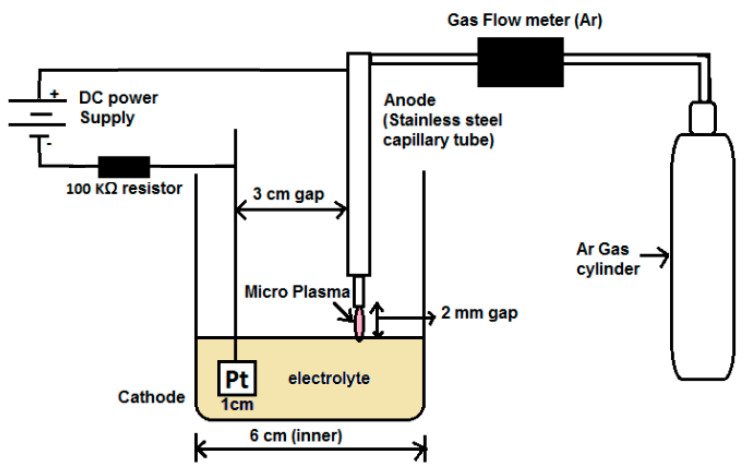
Schematic diagram of plasma–liquid interactions system employed to synthesize SF-AuNPs and DF-AuNPs [41].

In this study, a PLI setup with the liquid cathode (Figure 1) was employed to synthesize AuNPs. This PLI setup consisted of a reaction cell, high voltage DC-power supply (Gamma, model No. RR15−12r/220/M678), Hastings gas flowmeter for argon (model EALL−500P with a range from 0 to  1200 gm/min), and a gas cylinder (argon). The reaction cell was a glass beaker containing electrolyte solution and two electrodes. The first electrode was a platinum wire wound over a quartz slide (5 mm×5 mm) acting as a cathode and immersed in the solution while the second electrode was a stainless-steel capillary tube (outer diameter (OD = 1 mm) and internal diameter (ID = 0.2 mm)), placed 2 mm above the liquid surface acting as an anode. To avoid arcing in gas phase operation at high DC voltage, the separation between the platinum electrode and the capillary tube was set up to 3 cm. 

### 2.3. Synthesis and Characterization 

As the main reduction of metallic precursors in the PLI technique occurs at the plasma–liquid interface, suitable polarities of the electrodes were selected to push metallic precursors to this main reaction zone. For example, the liquid cathode was more suitable for cationic precursors while the liquid anode was appropriate for anionic ones. However, for the synthesis of gold nanoparticles from$\;HAuCl_{4}$, a liquid cathode case was more efficient even though one can use a liquid anode too [42]. This is because $HAuCl_{4}$ forms $AuCl_{4}^{-}$ in DI water, which is slightly less stable and separates into gold cations and chlorine anions according to the cyclic chemical reaction$\;AuCl_{4}^{-}\;⇄Au^{+3}+4Cl^{-}$ [43]. In the case of the liquid cathode, the$\;AuCl_{4}^{-}$ ions are pushed to the plasma–liquid interface where first gold cations $\left(Au^{+3}\right)$ are separated from $AuCl_{4}^{-}$ by the positive ions such as $H^{+}$ showered from the plasma and then reduced into the neutral gold atom $\left(Au^{0}\right)$ by other reducing agents such as a secondary electron. For the generation of atmospheric pressure plasma, argon gas flow rate of 50 SCCM was used. This plasma was then sustained at the operating current of 5 mA. 

Before starting each experiment, the platinum electrode and glass beaker were cleaned using aqua-regia (*HNO_3_ + 3HCl*) and then rinsed 5 times with *DI* water to confirm the removal of all possible impurities from their surfaces.

For the synthesis of SF−AuNPs, 20 mL electrolytic solution of gold precursor with 0.5 mM concentration was prepared in a glass beaker and then treated with atmospheric pressure microplasma for up to 25 min. Similarly, for the synthesis of DF−AuNPs, 20 mL solution consisting of 0.5 mM concentrations of gold precursors and 50 mM concentrations of DF was also treated with the same atmospheric pressure plasma for 25 min. These plasma-treated solutions were monitored with the help of UV−Vis absorption spectrometer to confirm the formation of gold nanoparticles and the completion of the synthesis processes. The as prepared SF−AuNPs and DF−AuNPs were subsequently stored in airtight glass vials for further characterizations and applications. 

To study the shape, size, morphology, and size distribution of SF−AuNPs and DF−AuNPs, scanning electron microscope (SEM) TESCAN MIRA3 was used. UV−VisSF−AuNPs

Before SEM analysis, DF−AuNP colloidal solutions of *SF-AuNPs* and *DF-AuNPs* were centrifuged for 10 min at 12,000 rpm. After removing the clear solution, the remaining precipitates were redispersed in the same amount of DI water and centrifuged again for 10 min at 12,000 rpm to confirm the removal of the remaining DF and gold precursors from AuNPs. For SEM imaging, the centrifuged SF−AuNPs and DF−AuNPs were redispersed in deionized water having 10 times smaller volume and then sonicated at room temperature for 30 min. Afterwards, 20 µL of the sonicated solutions of SF−AuNPs and DF−AuNPs were dried on two separate silicon slices at room temperature. In order to determine the elemental composition of SF−AuNPs and DF−AuNPs, energy dispersive X-ray (EDX) spectroscopy was used. In order to determine the crystalline structure of the as prepared SF−AuNPs and DF−AuNPs, X-ray Diffractometery () was carried out. For XRD analysis, Panalytical Xpert-Pro diffractometer was used. Fourier transform infrared (FTIR) spectroscopy was carried out using Shimadzu spectrometer (IRTracer-100) to ensure the successful coating of DF on the surfaces of AuNPs. For XRD and FTIR analysis, the centrifuged SF−AuNPs and DF−AuNPs were redispersed in deionized water of 20 times smaller volume to obtain a concentrated solution of SF−AuNPs and DF−AuNPs. For XRD analysis, 200 µL of this concentrated solution of both SF−AuNPs and DF−AuNPs was dried on glass substrates. Similarly, for FTIR analysis, 150 µL of the same concentrated solutions of DF−AuNPs was dried on a glass substrate. For FTIR spectroscopic reference analysis, 150 µL of DF solution was also dried on a glass substrate. 

### 2.4. SERS Analysis 

Raman spectroscopy was employed to study the SERS base trace molecular detection capability of SF−AuNPs and DF−AuNPs by using RAMBOSS Raman spectrometer by Dong Woo Optron, South Korea. For SERS analysis, the centrifuged solutions of both SF−AuNPs and DF−AuNPs were redispersed in DI water having 6 times smaller volume to make their concentrated solution. Firstly, the concentrated NP solutions of 20 µL were dried at room temperature onto a glass substrate after which 20 µL solutions (10^−6^ M) of R6G were dried on top of the dried layers of both SF−AuNPs and DF−AuNPs. For SERS reference analysis, 20 µL solution of R6G (1 µM) was dried on a glass substrate. Similarly, 20 µL solutions of SF−AuNPs and DF−AuNPs were dried on glass substrates for studying of their Raman spectra. To investigate the coverage of the exposed regions with SF−AuNPs and DF−AuNPs, optical microscope, integrated within the Raman system, was used. 

### 2.5. Catalysis Experiments 

For catalytic activity, the centrifuged solutions of both SF−AuNPs and DF−AuNPs were re-dispersed in DI water having the same volume. A 300 µL solution of NaBH_4_ (0.1 M) and 2.7 mL solution of MB (0.05 mM) were mixed in a cuvette. Subsequently, 50 µL solution of SF−AuNPs was added to the same cuvette containing the mixture of NaBH_4_ and MB. Afterwards, the degradation process of MB was monitored via UV−Vis absorption spectroscopy on regular bases of time intervals of 1 min. Similarly, to study the catalytic activity of DF−AuNPs, 2.7 mL MB (0.05 mM), 300 µL $NaBH_{4}$ (0.1 mM), and 50 µL DF−AuNPs solutions were mixed in a cuvette and then monitored for degradation processes with the help of UV−Vis absorption spectroscopy.

## 3. Results and Discussion 

### 3.1. Synthesis and Characterization of SF−AuNPs and DF−AuNPs

In the PLI setup with a liquid cathode, the positive ions such as argon ions ($Ar^{+}$), air ions$\;(N_{2}^{+},\;O_{2}^{+})$, and water vapor ions $\left({\left(H_{2}O^{+}\right)}_{g}\right)$ generated in the plasma region are showered on the liquid surface and they ionize or dissociate the water molecules into highly reactive species such as $H^{\pm },OH^{\pm },\;\;{\left(H_{2}O^{+}\right)}_{aq}\;\mathrm{and}\;e_{aq}^{-}$ [33,44]. These radicals are crucial for the reduction of the gold ions in the solution and convert them into neutral gold atoms. Another strong reducing agent, hydrogen peroxide $\left(H_{2}O_{2}\right)$, is also generated in the liquid due to the combinations of OH radicals that have a longer life as compared to other radicals. These radicals can also reduce gold precursors at the plasma–liquid interface as well as in the bulk liquid. The expected reduction reactions of the gold precursors in the solution by $e^{-},\;H^{-},\;OH^{-},$ and $H_{2}O_{2}$ are given bellow [34,45]:

(1) $$Au^{+3}+3e^{-}\to Au^{0}$$

(2) $$Au^{+3}+3H^{-}\to Au^{0}+3H$$

(3) $$Au^{+3}+3OH^{-}\to Au^{0}+3OH$$

(4) $$2Au^{+3}+3H_{2}O_{2}\to 2Au^{0}+6H^{+}+3O_{2}$$

The supersaturation of the Au atoms causes nucleation and subsequently the growth of AuNPs. In the case of DF−AuNPs, *DF* controls the growth kinetics of AuNPs and enhances their stability as well. 

**Figure 2:**
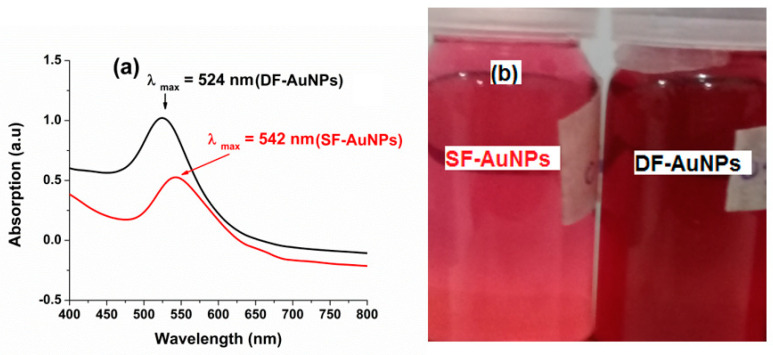
(**a**) UV-Vis absorption spectra of SF−AuNPs and DF−AuNPs; (**b**) Photos of SF−AuNPs and DF−AuNPs.

UV−Vis absorption spectroscopy is a proven technique to monitor both growth and formation of the nanoparticles of the desired materials, in terms of size and shape. As AuNPs have size- and shape-dependent signature SPR peaks, their formation and identification can be confirmed with the help of UV−Vis absorption spectroscopy. The UV−Vis absorption spectra of *SF* − *AuNPs* and DF−AuNPs (DF:Au=100:1) are given in Figure 2a, according to which the absorption peak of the DF−AuNPs appears narrower and blue-shifted as compared with that of the   SF−AuNPs, indicating the synthesis of smaller and relatively more uniform spherical AuNPs in the presence of the surfactant (DF). It is important to note that the change in the plasmon position of about 18 nm in this case cannot be simply due to the surface coating of DF−AuNPs. Although the presence of the surfactant changes the plasmon peak position due to the change in the local dielectric environment, the change is not that pronounced, i.e., 1 to 2 nm. On the other hand, the change in the size of the nanoparticles can cause a comparatively larger shift in the plasmon due to the phase retardation effects [46]. The change in the peak absorbance is also evident by the change in color of the two nanoparticle solutions shown in Figure 2b.

**Figure 3:**
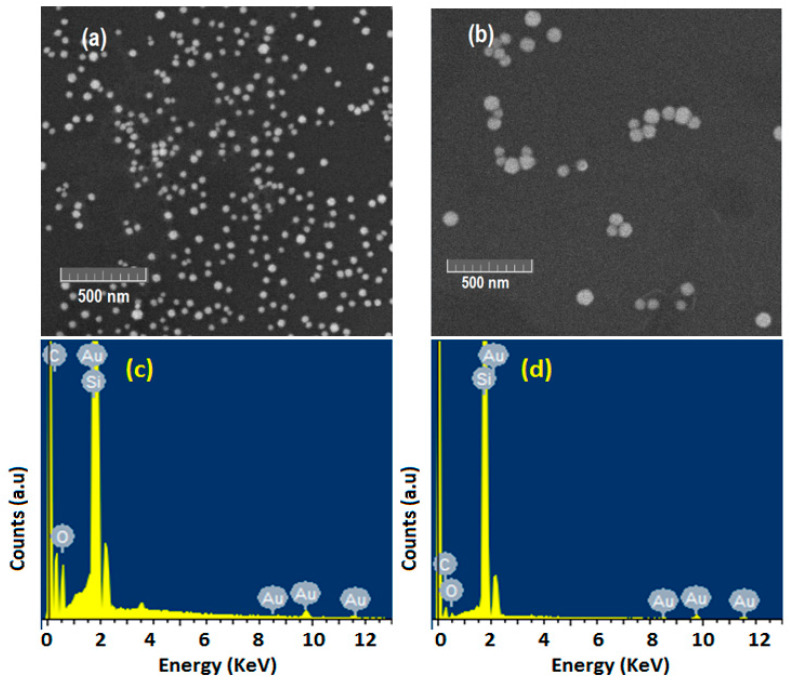
SEM images of (**a**) DF-AuNPs, (**b**) SF-AuNPs and EDX, (**c**) DF-AuNPs, (**d**) SF-AuNPs.

Figure 3a,b shows the SEM images of the DF−AuNPs and SF−AuNPs, respectively. In the presence of the surfactant (DF), the synthesized AuNPs are smaller, and relatively uniform spheres with an estimated average size of 20 nm (Figure 3a). However, in the absence of DF, l the synthesized AuNPs are relatively bigger with 40 nm as their estimated average size (Figure 3b). These SEM results and UV−Vis absorption spectra (Figure 2) are consistent [26].

The composition of elements in the as-synthesized DF−AuNPs and SF−AuNPs determined using EDX are given in Figure 3c and Figure 3d, respectively. As per elemental identification carried out via EDX spectrum, the sample comprises Au (due to *AuNPs*), carbon (likely due to *DF*, or due to burning during scanning), silicon (due to $SiO_{2}$ substrate on which DF−AuNPs and SF−AuNPs were deposited), and oxygen (due to *DF*).

**Figure 4:**
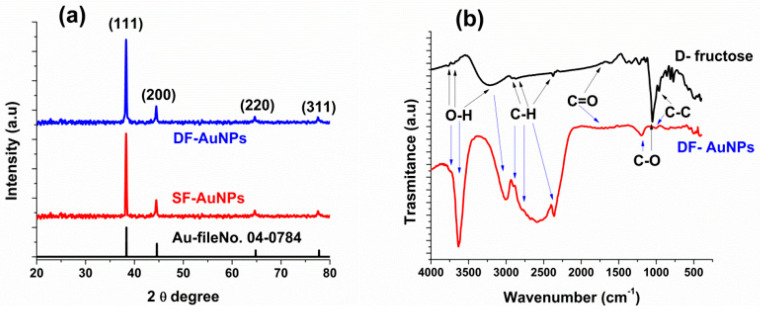
(**a**) XRD spectra of Au-file number 04-0784, SF-AuNPs, DF-AuNPs, (**b**) FTIR absorption spectra of DF and DF-AuNPs.

Figure 4a depicts the XRD patterns of SF−AuNPs and DF−AuNPs. In the figure, the Bragg’s peaks at 38.2°, 44.5°, 64.9°, and 77.9° correspond to the (111), (200), (220), and (311) planes of the face-centered cubic (FCC) crystalline structure of pure gold. The peaks match well with the reference card for gold JCPDS File number 04−0784 (black plot in Figure 4a). These XRD patterns confirm the crystalline nature of SF−AuNPs and DF−AuNPs. These XRD spectra also point to the formation of pure gold nanoparticles in the samples. 

In order to verify the functionalization of *AuNPs* with *DF*, *FTIR* spectroscopy of DF−AuNPs was carried out. The FTIR spectra of *DF* and DF−AuNPs are shown in Figure 4b. Vibrational bands of DF are classified as O−H vibrational stretching bands lying at 3876–3005 cm^−1^, C−H vibrational stretching bands at 3000–2061 cm^−1^, and C=O stretching bands at 1849–1634 cm^−1^. Furthermore, the characteristic bands related to DF including C−O and C−C stretching (900–1153 cm^−1^) lie at 1500–600 cm^−1^ [47,48]. In the FTIR spectra of DF (black line in Figure 4b), bands related to O−H stretching at 3771, 3707, and 3227 cm^−1^ and C−H vibrational stretching bands at 2927, 2870, 2374 cm^−1^ and 2334 cm^−1^ are seen. Moreover C=O, *C* − *O*, and C−C bands are observed at 1719 cm^−1^, 1051 cm^−1^, and 962 cm^−1^, respectively [33]. In the FTIR spectra of DF−AuNPs (red plot in Figure 4b), O−H bands at 3771 and 3707 cm^−1^ of DF are merged into a single strong, broadened and shifted band at 3634 cm^−1^ (lower wavenumber) () while the O-H band at 3227 cm^−1^ O−H becomes narrower and shifts to 3005 cm^−1^ (lower wavenumber) (), indicating the physisorption of *DF* via its *OH* functional groups with AuNPs. All C−H vibrational stretching bands are shifted to lower frequencies, i.e., 2927 to 2904 cm^−1^, 2870 to 2582 cm^−1^, and 2374 to 2349 cm^−1^. The C−H band at 2870 shifts to 2582 cm^−1^ and becomes stronger and broadened, indicating the binding of the hydroxyl group with AuNPs. All other remaining vibrational bands of *DF* are weakened and shifted upon AuNPs coordination. The observed broadening and shifting of the vibrational bands are expected due to the metal-ligand secondary bonding of the fructose’s hydroxyl groups with AuNPs, which affects the corresponding stretching vibrations. With the FTIR analysis of *DF* − *AuNPs*, it is confirmed that AuNPs are successfully coated with DF molecules.

### 3.2. SERSSF–AuNPsDF–AuNPs

**Figure 5:**
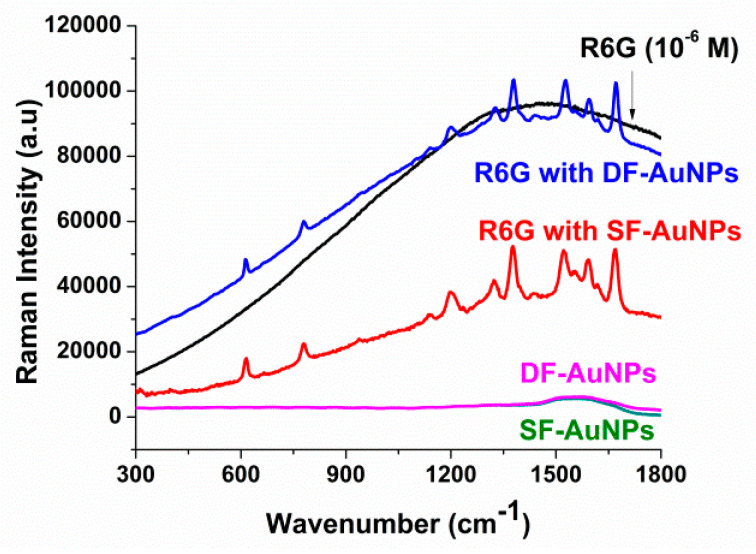
SRES spectra: black (R6G only), blue (R6G on DF-AuNPs), red (R6G on SF-AuNPs), pink (DF-AuNPs), and green line (SF-AuNPs).

The SERS spectra of R6G (µM), R6G deposited on SF−AuNPs and DF−AuNPs, and SF−AuNPs and *DF* − *AuNPs* without R6G are shown in Figure 5. The Raman spectra of both SF−AuNPs and DF−AuNPs in the absence of R6G are nearly horizontal lines with little humps between 1450 and 1720 cm^−1^, indicating no contributions to Raman scattering. The little humps in the Raman spectra of both SF−AuNPs and DF−AuNPs may be expected due to the glass substrates. When only R6G is deposited on the substrate, in the absence of SF−AuNPs and DF−AuNPs, a featureless broad hump appears as a result of the fluorescence of R6G. The laser with excitation wavelength of 514 nm is used which excites the fluorescence of R6G as well. As mentioned before, significantly higher fluorescence cross-section, as compared to the Raman cross-section, makes it difficult to observe Raman peaks under electronic excitation conditions [33]. However, when R6G is placed on the SF−AuNP or DF−AuNP substrate, several intense Raman peaks of R6G appear. In the SERS spectra of both SF−AuNPs and DF−AuNPs, the IP (in plane) ring band of C−C−C, OP (out of plane) and IP bands of C−H stretching at 661, 778 and 1137cm^−1^ are seen, respectively. The C−C aromatic stretching of R6G is seen at 1198, 1324, 1528, 1597, 1620, and 1670 cm^−1^, while that of C−N stretching is seen at 1379 cm^−1^. The peak positions agree well with the reported Raman spectra of R6G [41]. In the case of the SF−AuNPs substrate, the fluorescence of R6G is quenched, which is possible only if R6G molecules have a closer approach to the surface of SF-AuNPs. This fluorescence quenching also confirms the surfactant-free synthesis of AuNPs via our PLI setup. In fluorescence quenching, the energy from R6G is transferred to SF−AuNPs through a non-radiative decay process. The absence of fluorescence background makes Raman peaks more prominent. However, when the fluorophore is kept at a few nm from the surface of the nanoparticle, due to the surfactant (DF) that acts as a spacer, the fluorescence is not expected to quench [33]. 

This is exactly what we see in the SERS spectra of R6G on DF−AuNPs (blue curve, Figure 5). In this case, there is a clear presence of the fluorescence hump, but the Raman peaks of R6G are also visible on top of the fluorescent background. However, the intensity of the Raman peaks is relatively lower than that of SF−AuNPs. This is possible only due to the fact that in the presence of the surfactant (DF), R6G molecules must settle slightly away from the surface of the AuNPs, and, therefore, experience lowered field enhancement. Nonetheless, the Raman peaks are clearly visible on the top of the fluorescent background in this case as well, which verifies the excellent SERS enhancement capability of DF−AuNPs, even with the excitation source that excited the fluorescence of R6G.

### 3.3. Catalytic Properties of SF−AuNPs and DF−AuNPs

**Figure 6:**
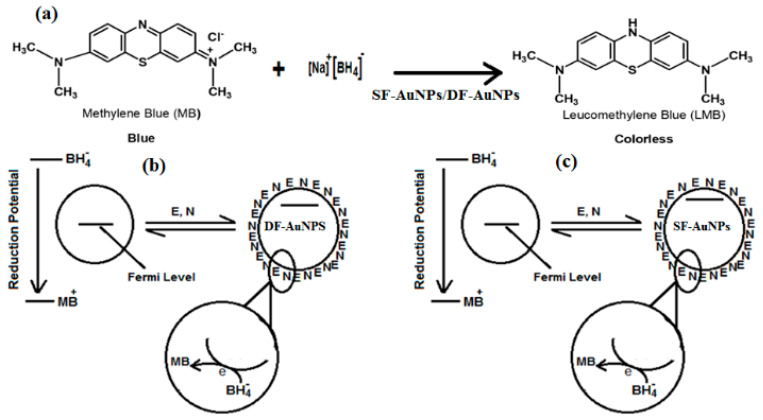
(**a**) reduction reaction of MB by SF-AuNPs/DF-AuNPs, and Schematic of catalytic process on (**b**) DF-AuNP, (**c**) SF-AuNP. E(electrophile, MB), N(nucleophile, $BH_{4}^{-}$ ions).

**Figure 7:**
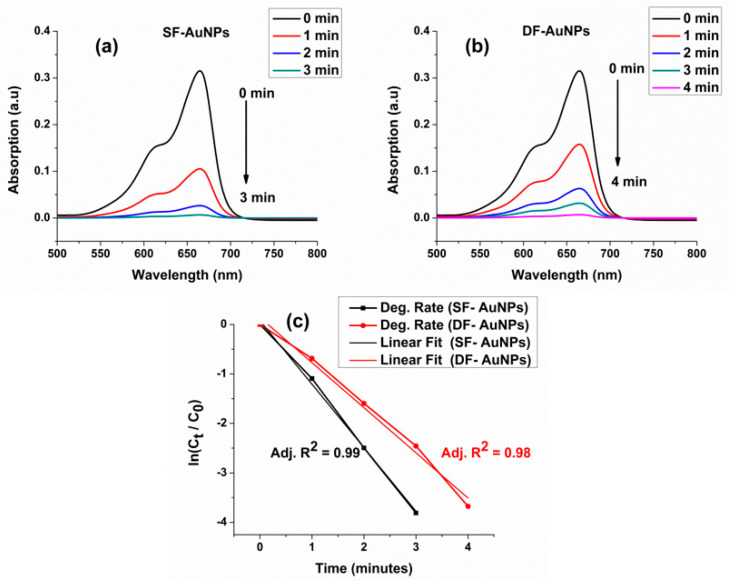
Catalytic degradation of MB by (**a**) SF-AuNPs, (**b**) DF-AuNPs, and (**c**) degradation rates.

Finally, the catalytic capability of both SF−AuNPs and DF−AuNPs is evaluated upon degradation of a toxic dye MB (blue color) into non-toxic and colorless leucomethylene blue (LMB). Figure 6 shows the reduction reaction (Figure 6a) of MB to LMB by $NaBH_{4}$ in the presence of catalysts ( SF−AuNPs and DF−AuNPs) and a schematic of the catalytic process at the surfaces of DF−AuNP (Figure 6b) and SF−AuNP (Figure 6c). Fermi levels of both SF−AuNPs and DF−AuNPs are lowered, i.e., negatively shifted when donor$\;BH_{4}^{-}$ ($BH_{4}^{-}$ ions) and acceptor (MB) are adsorbed on their surfaces. This increases the potential difference between MNPs and MB, which makes the reduction process of MB by $BH_{4}^{-}$ ions on the surfaces of both SF−AuNPs and DF−AuNPs easier and faster. In the case of SF−AuNPs, the adsorption of donor and acceptor on the surface of the particle is easier and closer due to the absence of any surfactant on their surfaces. Consequently, the catalytic capability of SF−AuNPs will be enhanced. To monitor the catalytic activity of SF−AuNPs, we put their suspension (50 µL) in a cuvette containing a solution of MB (2.7 µL) and $NaBH_{4}$ (300 µL) and monitor this reduction processes via *UV* − *Vis* absorption spectroscopy. The peak intensity of *MB* gradually decreases with the reaction time and completely decays within 3 min. Similarly, to study the degradation capability of DF−AuNPs, we again add a 50 µL solution of DF−AuNPs to a cuvette containing a solution of MB (2.7 mL) and $NaBH_{4}$ (300 µL) and monitor this degradation process with the help of UV−Vis absorption spectroscopy. In this case, the degradation is completed in 4 min, as depicted in Figure 7b. The degradation rate is calculated using ln(*C_t_/C_o_*) where *C_o_* and *C_t_* represent the absorbances at *t = 0* and *t = t*, respectively. A good linear fit to the graph (Figure 7c) indicates that the reaction follows pseudo first-order kinetics. SF−AuNPs and DF−AuNPs play the role of the mediator in the transferring of electrons from donor$\;BH_{4}^{-}\;$to MB for the reduction process. In the cases of SF−AuNPs and of DF−AuNPs, the rate constants of catalytic degradation are 1.284 min^−1^ and 0.912 min^−1^, respectively. This is lower than the other reported degradation rates for surfactant-free and surfactant-coated nanoparticles [49]. The higher degradation rate constant of SF−AuNPs compared to DF−AuNPs confirms the fact that the surfactant affects the catalytic properties of MNPs. The metallic surface reduces the bond dissociation energy (BDE) slightly more when both dye (MB) and $BH_{4}^{-}$ ions are adsorbed on its surface, which makes the transfer of electrons from donor to acceptor more efficient. Therefore, the catalysis by SF−AuNPs is more efficient as compared with DF−AuNPs. This is despite the fact that SF−AuNPs have a bigger size compared with DF−AuNPs, and it is well known that the catalytic activity of Au nanoparticles decreases with increasing particle size. Fenger et al. carried out a study of the comparative catalytic properties of CTAB-capped Au NPs of different sizes grown through the seeded method [50]. They reported that the apparent rate of reaction was 3 and 60 times larger when 13 nm NPs were compared with 28 nm and 56 nm Au NPs, respectively. This verifies that the faster catalytic activity of SF−AuNPs compared to DF−AuNPs is due to the absence of surfactant coating. 

On the other hand, a higher degradation rate for DF−AuNPs compared to other reported results of degradation of surfactant-coated AuNPs could be due to the relatively smaller size of the DF molecule. For example, the size of the bilayer of CTAB, which is commonly used as a stabilizer in the wet-chemical synthesis of gold nanoparticles, is about 4 nm [51], which is almost twice the size of the *DF* molecule [47]. 

## 4. Conclusions 

Fast, efficient, cost-effective, pure, and eco-friendly synthesis of SF−AuNPs and DF−AuNPs was carried out using the plasma liquid interaction (PLI) technique. Different diagnostic techniques were utilized to monitor the morphology, structural, sensing, and catalytic abilities of the as synthesized gold nanoparticles. X-ray Diffractometery and EDX analysis confirmed the purity of both *SF-* and *DF-AuNPs*. The SERS and catalytic capabilities of both *SF-* and *DF-AuNPs* were demonstrated. In particular, the SERS-based trace detection of the rhodamine-6G (R6G) molecule and catalytic degradations of methylene blue (MB) were tested. This analysis confirms that both SF−AuNPs are superior to DF−AuNPs, both for catalysis and SERS-based trace detection. A promising enhancement in the Raman signal of R6G was observed for both types of particles but Raman peaks were much more prominent due to the quenching of the fluorescence background signal of R6G for SF−AuNPs. Moreover, SF−AuNPs showed superior catalytic degradation capability, as compared to the DF−AuNPs, due to their uncapped surfaces, which made the absorption of dye and reducing agent easier on their surface. Nonetheless, due to the non-toxicity, high stability, and smaller size of *DF* molecules, DF−AuNPs are excellent candidates for replacing AuNPs stabilized either with toxic or larger molecular surfactant, especially for sensing, catalytic, and biological applications. 

## Data Availability

The data that support the findings of this study are available from the corresponding author upon reasonable request.
